# Genetic Dissection of Seasonal Changes in a Greening Plant Based on Time-Series Multispectral Imaging

**DOI:** 10.3390/plants12203597

**Published:** 2023-10-17

**Authors:** Taeko Koji, Hiroyoshi Iwata, Motoyuki Ishimori, Hideki Takanashi, Yuji Yamasaki, Hisashi Tsujimoto

**Affiliations:** 1 The United Graduate School of Agricultural Sciences, Tottori University, 4-101 Koyamacho Minami, Tottori 680-8553, Japan; tkoji0305@gmail.com; 2 Graduate School of Agricultural and Life Sciences, The University of Tokyo, 1-1-1 Yayoi, Bunkyo, Tokyo 113-8657, Japan; iwata@ut-biomet.org (H.I.); ishimori@ut-biomet.org (M.I.); atakana@g.ecc.u-tokyo.ac.jp (H.T.); 3 Arid Land Research Center, Tottori University, 1390 Hamasaka, Tottori 680-0001, Japan; yujyamas@g.ecc.u-tokyo.ac.jp

**Keywords:** multispectral analysis, phenotyping, QTL analysis, seasonal change, morphological change, vegetation index, dryland

## Abstract

Good appearance throughout the year is important for perennial ornamental plants used for rooftop greenery. However, the methods for evaluating appearance throughout the year, such as plant color and growth activity, are not well understood. In this study, evergreen and winter-dormant parents of *Phedimus takesimensis* and 94 F_1_ plants were used for multispectral imaging. We took 16 multispectral image measurements from March 2019 to April 2020 and used them to calculate 15 vegetation indices and the area of plant cover. QTL analysis was also performed. Traits such as the area of plant cover and vegetation indices related to biomass were high during spring and summer (growth period), whereas vegetation indices related to anthocyanins were high in winter (dormancy period). According to the PCA, changes in the intensity of light reflected from the plants at different wavelengths over the course of a year were consistent with the changes in plant color and growth activity. Seven QTLs were found to be associated with major seasonal growth changes. This approach, which monitors not only at a single point in time but also over time, can reveal morphological changes during growth, senescence, and dormancy throughout the year.

## 1. Introduction

Perennial ornamental plants change their appearance, including leaf color, as they go through growth, senescence, and dormancy. Vegetation indices are mathematical formulae that use the ratio of different wavelengths of light reflected by plants to estimate various vegetation characteristics [1]. The normalized difference vegetation index (NDVI) is one of the vegetation indices [2] and reflects plant activity and cover. For example, in many deciduous trees, NDVI values increase rapidly with the breaking of dormancy in spring, peak in early summer, decrease in fall, and reach a minimum during dormancy [3,4]. Winter dormancy is triggered by shorter days. The accumulation of low temperatures and the change to longer days cause dormancy breaking, which is followed by flowering in many temperate trees [5]. Phloem capacity for signal and carbon movement in the dormancy break period is involved in growth at this time [6]. Many plant species, such as wheat and sorghum, senesce after the growth season [7,8,9,10,11], and their appearance changes accordingly. Changes in the cover area of street trees and other urban greening plants with seasons and seasonal color changes were reported from a landscape perspective [12,13]. However, the good appearance of perennial ornamental plants is important all year round; thus, the evaluation of seasonal morphological changes in such plants throughout the year is needed.

Seasonal color changes are evaluated with color charts or color readers. Evaluation with a color chart is non-destructive and simple, but objective evaluation is difficult [14]. Because color usually varies throughout the plant body, judging the color at only one point is insufficient, and color readers have difficulty determining a representative measurement point [15,16]. Recently, vegetation indices calculated from wavelength values from multispectral imaging have been used to measure seasonal changes in plant activity with high throughput [7,9,10,17,18,19]. Cameras for multispectral imaging are capable of capturing a plant body in a plane rather than at a single point, and each of the reflected multiple wavelengths of visible and invisible light can be measured separately. Therefore, this approach seems to be suitable for evaluating the color and other appearance factors of ornamental plants, but few examples of such applications have been reported.

*Phedimus takesimensis* (Crassulaceae) is used for rooftop greenery in urban areas in Japan. *P. takesimensis* is an ornamental plant; a low degree of dormancy and unfurled leaves in winter are preferred from the standpoint of appearance. *Phedimus* species grow after a dormancy break in spring, are deciduous in fall, and many of them overwinter with dormant buds because of their high degree of dormancy [20,21]. Because of these characteristics and the importance of good appearance for ornamental plants, it is important to evaluate the changes in leaf coloration with seasonal changes in *Phedimus* species. Using multispectral image analysis, we previously evaluated plant color, area of plant cover, and 15 vegetation indices in 94 F_1_ plants derived from a cross between two parental lines (evergreen P_1_ and winter-dormant P_2_) of *P. takesimensis* [20]. We demonstrated that this approach allows for quantitative evaluation and genetic analysis of the plant color and degree of dormancy break [20]. However, we analyzed only one seasonal time point (two measurement time points), namely, in April 2019 and April 2020.

The purpose of this study was to monitor seasonal changes in *P. takesimensis* appearance using multispectral image analysis throughout the year in the F_1_ population established in [20] and to perform a genetic analysis of these changes. We constructed a quantitative time-series system for evaluating plant color and vegetation indices. Using QTL analysis, we identified the genetic loci involved in changes in appearance throughout the year. We demonstrated the effectiveness of multispectral analysis and genetic analysis based on [20] in the analysis of seasonal color changes of this ornamental plant.

## 2. Results

### 2.1. Changes in Vegetation Indices and Area over Time

**Figure 1:**
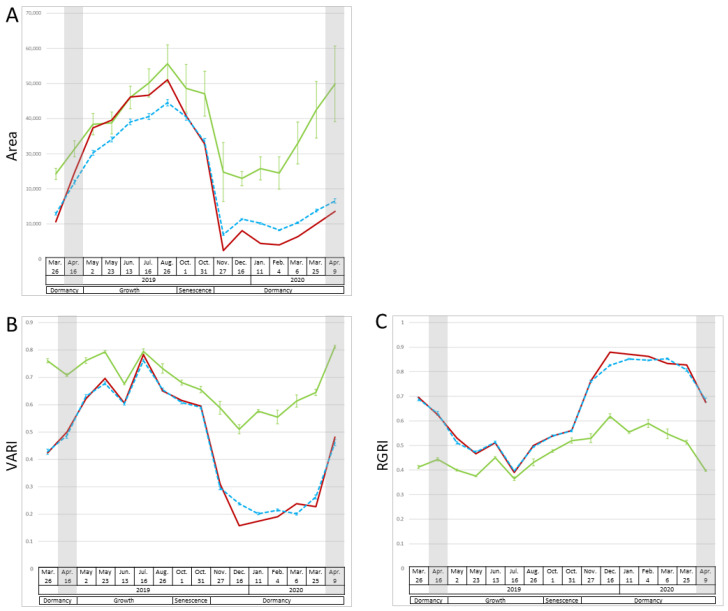
Seasonal changes in the area of plant cover, VARI, and RGRI. (**A**) Area of plant cover, (**B**) VARI, and (**C**) RGRI. Green line, mean of P_1_ (*n* = 4); red line, P_2_ (*n* = 1); blue dashed line, mean of F_1_ (*n* = 94). Error bars, standard errors. Gray shading, the dates used in [20].

We took 16 multispectral image measurements from March 2019 to April 2020 and used them to calculate 15 vegetation indices and the area of plant cover to assess morphological changes throughout the year, such as growth, senescence, and dormancy in the P_1_, P_2_, and F_1_ populations (Figure 1 and Figure S1). Two patterns of changes in index values were detected. The first was an increase from spring to summer, followed by a decrease in fall and low values in winter and early spring. In winter and early spring, the values were higher in P_1_ than in P_2_ and F_1_. The pattern of an increase and decrease in the area of plant cover corresponded to this pattern (Figure 1A). Vegetation indices related to biomass (visible atmospherically resistant index (VARI), NDVI, simple ratio (SR), enhanced vegetation index (EVI), visible atmospherically resistant indices green (ViGreen)), plant pigments (structure insensitive pigment index (SIPI), pigment-specific normalized difference (PSND)), chlorophyll (chlorophyll absorption ratio index (CARI), modified chlorophyll absorption ratio index (MCARI)), and carotenoids (carotenoid reflectance index 1 (CRI1)) also fell within this pattern (Figure 1 and Figure S1). The second pattern was a decrease from spring to summer, followed by an increase in fall and high values in winter and early spring. The index values of P_1_ were not as high as those of P_2_ and F_1_ in winter and early spring. The vegetation indices related to anthocyanins (red–green ratio index (RGRI) and anthocyanin reflectance index (ARI)) corresponded to this pattern (Figure 1C and Figure S1). The other three vegetation indices (anthocyanin content index (ACI), carotenoid reflectance index 2 (CRI2), and photochemical reflectance index (PRI)) did not show a clear pattern of changes. The values of each vegetation index, especially in winter, of F_1_ tended to be closer to those of P_2_ than to those of P_1_. For example, P_1_ had higher VARI values than P_2_ and F_1_ during the period from March to April 2019 and November to April 2020 (at *p* ≤ 0.001), while the VARI values did not differ significantly (at *p* > 0.05) between P_1_, P_2_, and F_1_ during the period from May to August 2019.

### 2.2. Trends in Temporal Changes Found Using PCA

**Figure 2:**
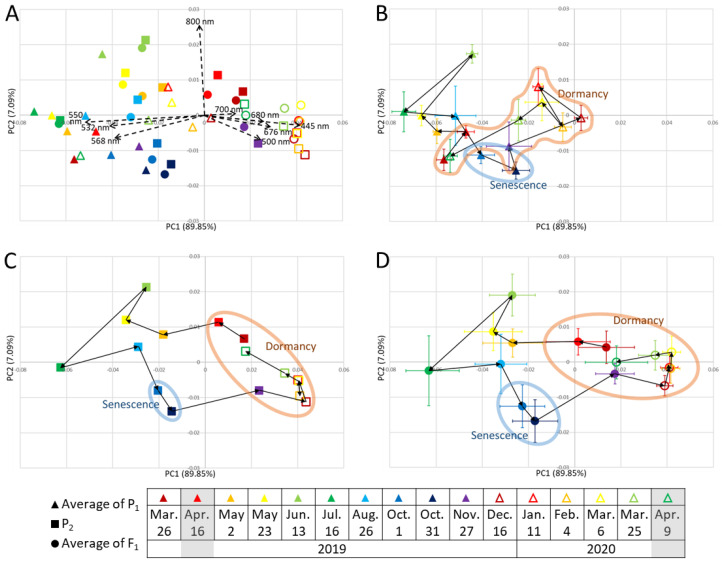
Changes in principal components (PCs) over one year. Triangles, means of P_1_ (*n* = 4); squares, P_2_ (*n* = 1); circles, means of F_1_ (*n* = 94). Error bars, standard deviations. Measurement dates are color-coded as shown in the figure key. (**A**) All values of P_1_, P_2_, and F_1_. Each black dashed arrow indicates a PC. (**B**) Means of P_1_. (**C**) P_2_. (**D**) Means of F_1_. Black solid arrows in (**B**–**D**) connect the data points in the order of the measurement dates. Data points enclosed in colored shapes: senescence period, October 1 to 31; dormancy period, November to April. Gray shading in the key, dates used in [20].

Principal component 1 (PC1) contributed almost 90% and explained the difference between the reflection of green light (532, 550, and 568 nm), and those of blue (445 nm), green (500 nm), and red light (676, 680, 700 nm) (Figure 2). The PC1 scores were the highest from December to March, when plants tended to reflect more blue and red light. PC1 scores were the lowest in July, when plants tended to reflect green light more. PC2 contributed 7% and explained the high or low reflection of near-infrared light at 800 nm. The PC2 scores were the highest in June, when plants tended to reflect more 800 nm light, and were the lowest on 31 October, when plants tended to reflect less 800 nm light.

The PCA plot positions were approximately the same on 25 March 2019 and 19 April 2020 but moved considerably throughout the year according to growth, senescence, and dormancy. PC1 values decreased in March and April with dormancy breaking. PC1 values increased in November for P_2_ and F_1_ and in December for P_1_ and were high during the winter months, with little variation. The pattern of changes in plot positions was similar for P_2_ and F_1_; their difference from P_1_ was particularly clear during the dormancy period, when their PC1 values tended to be higher than that of P_1_.

### 2.3. Correlation between Traits and Measurement Date

**Figure 3:**
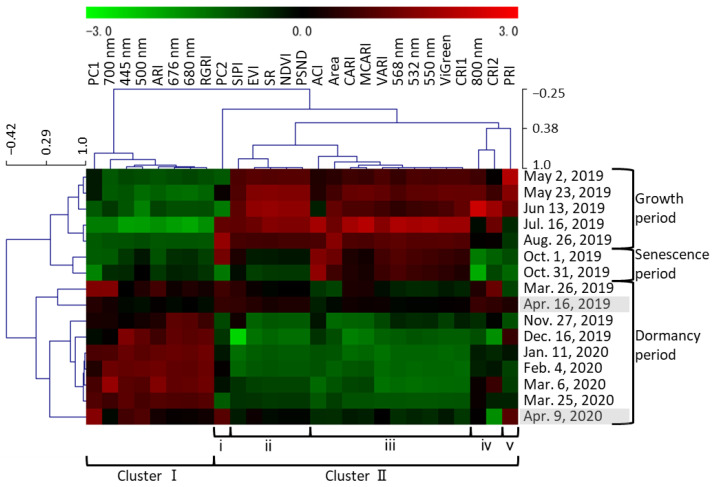
Correlations between all traits and all measurement dates. Each cell represents the mean value of all F_1_ individuals. All 27 traits (area of plant cover, 9 wavelength values, PC1 and PC2 values, and 15 vegetation indices) are listed at the top along the horizontal axis. Roman numerals at the bottom indicate the cluster classification of traits. All 16 measurement dates are listed along the vertical axis; gray shading indicates the dates used in [20].

All 27 traits (area of plant cover, reflectance at nine wavelengths, PC1 and PC2 scores, and 15 vegetation indices) were clustered on the basis of the mean value of all F_1_ individuals on each date (Figure 3). The clustering of all 16 dates generally followed the date order, and the dates that were close in time were closely related to each other. The clusters of dates were roughly classified into the growth and senescence period (May to October) and the dormancy period (November to April). The former was further divided into the growth period (May to August) and the senescence period (October). The 27 traits were divided into two clusters. Cluster I traits tended to have positive values during dormancy. This cluster included vegetation indices related to anthocyanins (excluding ACI), PC1 scores, and red light (which positively affected PC1 scores). Cluster II traits tended to have positive values in the growth and senescence periods. This cluster included the area of plant cover and vegetation indices related to biomass, plant pigments, chlorophyll, carotenoids, and light use efficiency. It also included green light, which negatively affected PC1 scores, and near-infrared light, which positively affected PC2 scores. Cluster II was further divided into subclusters i through v. Only PC2 was classified in subcluster i; the pattern of PC2 changed differently from those of the other traits; in particular, it had positive values in July, in August, and on 1 October. Traits classified in ii and iv tended to have positive values during the growth period and negative values during the senescence period. Subcluster ii included NDVI, SR, EVI, SIPI, and PSND. Traits in subcluster iii, such as VARI and ViGreen, tended to have positive values in both the growth and senescence periods. Subcluster iv included CRI2 and reflectance at 800 nm; the latter was the highest in June and the lowest on 31 October. Subcluster v included only PRI; during the growth period, it was positive until June and turned negative in July.

### 2.4. QTLs Detected in Different Seasons

**Figure 4:**
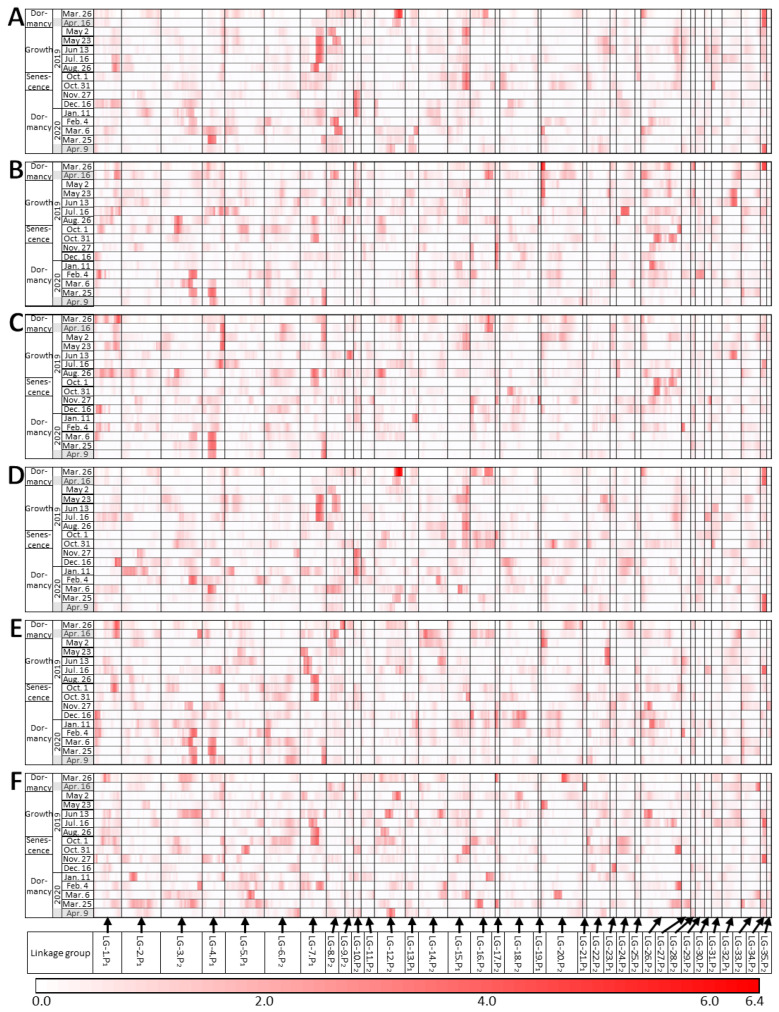
Changes in QTL results for traits over time. (**A**) PC1, (**B**) PC2, (**C**) NDVI, (**D**) VARI, (**E**) 800 nm, and (**F**) PRI. Dates of measurements are listed along the vertical axis; the positions of chromosomal markers are shown along the horizontal axis. Darker red indicates higher LOD values. Gray shading, the dates used in [20].

The QTL analysis was performed for all 27 traits. We focused on the QTLs detected on at least two dates and found seven such QTLs (Figure 4, Table S1). Three QTLs were found at the end of the dormancy period. The first QTL was found in LG-34.P_2_ for 4 traits on 26 March and 10 traits on 16 April 2019, and for 3 traits on 25 March and 8 traits on 9 April 2020. The QTLs for VARI (Figure 4D), ViGreen, ARI, RGRI, and CRI1 were detected on multiple dates. Of all the QTLs detected in March and April, only this QTL was found in both years. The second QTL was found in LG-4.P_1_ for five traits on 25 March and for two traits on 9 April 2020. The QTLs for NDVI (Figure 4C), SR, SIPI, and PSND were significant at the 5% level. The third QTL was found in LG-12.P_2_ for nine traits on 26 March and for one trait on 16 April 2019. The QTLs for VARI (Figure 4D), ViGreen, PC1 (Figure 4A), CARI, and RGRI were significant at the 5% level.

One QTL in LG-20.P_2_ was found on 26 March 2019 (end of dormancy), and on 2 May 2019 (beginning of growth). A PC2 QTL was detected on both dates (Figure 4B). QTLs for 800 nm (Figure 4E) and EVI were detected on 2 May. All three QTLs detected on 2 May were significant at the 5% level. One QTL in LG-7.P_1_ was found on 23 May for four traits and on 13 June for five traits (both dates were during the growth period). The QTLs for CRI1 and two wavelength values were significant at the 5% level. One QTL in LG-15.P_1_ was found for five traits on 2 May and for four traits on 26 August (during growth). It was also found for four traits on 1 October and for one trait on 31 October (senescence). QTLs for PC1 (Figure 4A) and VARI (Figure 4D) were detected on several dates. One QTL in LG-10.P_2_ was found for eight traits on 27 November and for three traits on 11 January (beginning of dormancy). A QTL for PC1 (Figure 4A) was detected on both dates. QTLs for PC1, VARI (Figure 4D), ViGreen, and RGRI were significant at the 5% level on 27 November.

These QTLs tended to be found when seasonal changes occurred, such as dormancy breaking, growth, senescence, and the beginning of dormancy. Three out of seven QTLs were observed from late March to April (dormancy breakthrough). Fewer QTLs were observed from February to 6 March 2019 (dormancy) than during any other time of the year (Table S1). Even when the significance was marginal (less than 10% level), the LOD values tended to be relatively high on several consecutive dates, for example, for PC2 in LG-3.P_2_ on 26 August and 1 October (Figure 4B) and NDVI in LG-4.P1 on 16 April, 2 May, and 23 May (Figure 4C). Including all QTLs that were not observed over multiple measurement days, the highest number of QTLs among all dates was found for 676 nm (nine QTLs in total) at 5% and 10% significance (Table S1), followed by CRI1 and PC1 (eight QTLs) and 568 nm, VARI, and ViGreen (seven QTLs). In many cases, an identical QTL was seen for more than one trait, and multiple traits with an identical QTL on a given date were highly correlated in the clustering (Figure 3 and Figure S2).

## 3. Discussion

In this study, multispectral image analysis was extended to monitor changes in the perennial ornamental plant *P. takesimensis* throughout the year. This monitoring, rather than undertaking one seasonal time point (two measurement time points) analysis [20], allowed us to assess seasonal changes. The vegetation indices helped us to quantitatively evaluate the changes. The PCA of all wavelengths and vegetation indices enabled us to gather detailed information on the growth activity and color changes associated with seasonal changes in plant morphology, which could not be obtained from the vegetation index information alone. Seasonal QTLs were found. Thus, our proposed approach captured the seasonal variation in this perennial species.

### 3.1. Seasonal Changes during the Year

#### 3.1.1. Growth Period (May to August)

In addition to the area of plant cover, the values of vegetation indices related to biomass, such as NDVI and VARI, increased during the spring, and many of them peaked in June or July (Figure 1, Figures S1 and S3). NDVI indicates the photosynthetically active biomass of the plant canopy [2] and is widely used to assess plant health and growth activity [19]. The NDVI of many plants, such as wheat and poplar, greatly increases during vigorous plant growth [3,11]. VARI was used to investigate growth activity and vegetation in corn and mangrove forests [19,22]. This study’s results are consistent with previous studies in that NDVI and VARI tended to increase during the growth period. PC2 described the high or low reflection of near-infrared light at 800 nm (Figure 2); the reflection is related to the leaf cellular structure [23]. The 800 nm reflectance is higher in healthier and more active plants [24,25]. Therefore, we inferred that PC2 reflected growth activity. PC2 scores peaked in June and then decreased (Figure 2). These vegetation indices and PC2 results indicated vigorous growth during the spring after dormancy, with a peak around June, followed by a decline in growth activity.

#### 3.1.2. Senescence Period (October 1 and 31)

The values of traits belonging to cluster II (Figure 3), such as the vegetation indices for biomass, tended to decrease during senescence (Figure 1, Figure 3 and Figure S1). PRI was positive until June, but negative in July (Figure 3); it correlates with the ratio of chlorophyll to carotenoids and is used as an indicator of photosynthetic efficiency [26]. These results show that growth peaked in June and declined with aging, with the lowest growth activity on 31 October, just before defoliation (as visually determined) (Figure S3). The values of traits in subcluster ii were positive during growth but negative during senescence (Figure 3). Among the vegetation indices related to biomass, NDVI, SR, and EVI are calculated using 800 nm data and were classified in this subcluster; they may reflect the degree of leaf senescence just before defoliation. The values of traits in subcluster iii (Figure 3) were positive during growth and senescence. Among the vegetation indices related to biomass, VARI and ViGreen, which are calculated without 800 nm data, were in subcluster iii. They remained high even during senescence, suggesting that they do not reflect aging. NDVI decreases during aging in deciduous trees, sorghum, and wheat [4,7,9,10,11]. EVI and VARI reflect the aging of sorghum [7,8]. Although both of these vegetation indices reflect plant activity, in this study, vegetation indices, such as NDVI and EVI, which used 800 nm in the calculations in this study, reflected aging better. The PC2 score, which reflected the 800 nm data, declined after June and again after August, with a minimum on 31 October (Figure 2). The 800 nm value reached a maximum in June and a minimum on 31 October (Figure 3), which was consistent with the change in PC2 (Figure 2). The 800 nm reflectance values and the vegetation indices, such as NDVI, SR, and EVI, which use the 800 nm values in their calculations, could reflect aging well. SIPI and PSND, which reflect the ratio of carotenoids to chlorophyll and the amount of carotenoids, respectively, also use 800 nm values in their calculations; they were also classified in subcluster ii and were also inferred to reflect aging.

#### 3.1.3. Dormancy Period (November to April)

Beginning of dormancy period due to defoliation (November to January): Most of P_2_ and F_1_ completed defoliation in November and P_1_ in December (Figure S3). Since PC1 explained the reflection of visible light (Figure 2), we assumed that it represented plant color. PC1 values increased considerably in November for P_2_ and F_1_, and in December for P_1_. At that time, defoliation was completed and new reddish-weak-greenish shoots became exposed (Figure S3). In deciduous trees, NDVI values decrease rapidly in autumn and become very low in winter when the leaves are gone [4]; the same trend was observed here. The trends of the mean F_1_ values (Figure 3) also changed considerably after November, indicating the beginning of the dormancy period.

Dormancy period and degree of dormancy (February to early March): The PC1 scores of P_1_, P_2_, and F_1_ were the highest during the dormancy period and were particularly high in P_2_ and F_1_ (Figure 2). This reflected reddish or brownish leaves in winter (Figure S3). Many plant species, such as *Galax urceolata* and *Mikania micrantha*, accumulate anthocyanins in response to low winter temperatures and turn red [27,28], and it was similar to the plant color change in this study. Consistently, the values of RGRI and ARI were high during the dormancy period, especially in P_2_ and F_1_ (Figure 1 and Figure S1). The PCA plot positions did not change considerably during winter (Figure 2), which was consistent with fewer changes in color and morphology, as PC1 and PC2 reflected plant color and 800 nm reflection (i.e., plant activity), respectively. We speculated that the lowest number of QTLs during February and early March was due to less morphological change. P_2_ and F_1_, but not P_1_, had similar trends in each vegetation index and wavelength value (Figure 1, Figure 2 and Figure S1). These results suggest that F_1_ was more closely related to P_2_ than to P_1_. These results also shed light on the differences in seasonal change patterns due to genetic differences. These differences between P_2_ and P_1_ and between F_1_ and P_1_ were particularly pronounced during the dormancy period, and each value during this period was assumed to reflect the degree of dormancy.

P_1_ had higher values than P_2_ and F_1_ of vegetation indices related to biomass, plant pigments, chlorophyll content (CARI and MCARI), and carotenoids (CRI1). CARI and MCARI estimate photosynthetic activity [29]; in wheat and maize, higher chlorophyll content is associated with the ability for maximum growth [30], and a similar trend was shown in this study. These results presumably reflected higher growth activity of P_1_ during this period in comparison with P2 and F1. P_1_ had the lowest values of RGRI and ARI (Figure 1 and Figure S1). Because anthocyanin contents increase during winter dormancy in response to low temperatures [27,28], this result confirms that P_1_ was less dormant than P_2_ and F_1_, consistent with its green color. Cedar accumulates a type of carotenoid that protects it from excessive light during winter [31]. Higher CRI1 values indicate higher concentrations of carotenoids than of chlorophyll. The CRI1 values were higher in P_1_ than in P_2_ and F_1_ during the dormancy period (Figure S1). P_1_, whose leaves are expanded in winter, may respond to winter light stress by accumulating carotenoids.

Few studies have been conducted on the evaluation of the degree of dormancy of *Phedimus* species, including *P. takesimensis* [20]. The vegetation index data obtained in this study may provide a quantitative evaluation of the degree of dormancy.

Segregation of F_1_: The F_1_ population varied in leaf color, leaf size and shape, and plant height, but its basic morphology was closer to that of P_2_. Winter dormancy was also high, similar to that of P_2_. Although the genetic backgrounds of P_1_ and P_2_ are unclear, we assumed that the low dormancy of P_1_ was a recessive homozygous trait, while the high dormancy of P_2_ was a dominant homozygous trait. If so, self-pollination or mating between F_1_ siblings could result in individuals with low dormancy in the next generation.

Breaking dormancy (late March to April): From late March to April, the PC1 scores decreased considerably (Figure 2), indicating that the red leaf color changed to green, consistent with the previous results [20] (Figure S3). The vegetation index for biomass, such as NDVI, began to increase (Figure 1 and Figure S1), consistent with the reports of a sharp increase in NDVI during dormancy breakthrough in deciduous trees [3,4]. The vegetation index of anthocyanins, such as RGRI and ARI, began to decrease (Figure 1 and Figure S1), indicating a transition to the growing period. The genes involved and the mechanisms of breaking dormancy after the low-temperature period in winter were reported for rosaceous woody plants, such as Japanese apricot [32], and it is possible that a similar mechanism may have broken dormancy in this study. We detected the highest number of QTLs during this time and assumed that this abundance of QTLs could be explained by the large morphological changes. The QTL on LG-34.P_2_ may be related to the early dormancy breaking [20]. The number of traits affected by this QTL was the largest in this study and only this QTL was detected over the 2-year period; therefore, this QTL was particularly influential.

### 3.2. Small Effect of Each QTL

We speculated that the timing of morphological changes in the F_1_ population varied between individuals, which was manifested as differences in color and growth. The effects of most QTLs appeared to be small, and visual differences between F_1_ individuals were unclear.

Several chromosomal regions had high LOD values on multiple dates, although with marginal significance (less than 10% level), suggesting that these regions may be QTLs. For example, regarding PC2, such a region was found in LG-3.P_2_ from August (end of growth) to 1 October (beginning of senescence) (Figure 4B), when the PC2 score decreased (Figure 2). Therefore, this chromosomal region may be a QTL related to aging. Many small-effect QTLs affect the phenotype in rice, wheat, and ryegrass [33,34,35]. QTLs with marginal significance, such as those found in this study, are often overlooked when analyzed at a single time point. The temporal analysis in this study suggests the existence of multiple QTLs with small effects.

While the breeding of *P*. *takesimensis* has not progressed, by aggregating many such small QTLs, it may be possible to select individuals close to the desired trait in the future.

### 3.3. Morphological Changes Were Repeated Annually

The initial PCA plot positions for P_1_, P_2_, and F_1_ on 26 March 2019, moved over time and returned by 9 April 2020, when the measurements were completed (Figure 2). This suggests some degree of reproducibility of our data and that PCA can successfully represent seasonal changes year by year in perennial plants.

## 4. Materials and Methods

A detailed description of the materials and methods can be found in Koji et al. [20].

### 4.1. Plant Materials

*Phedimus takesimensis* ‘Tottori Fujita 1’ (Fujita Co., Ltd., Iwami-cho, Tottori Pref., Japan) was used as parent 1 (P_1_) and *P. takesimensis* collected in Gwacheon-si (Gyeonggi-do, Korea) was used as parent 2 (P_2_). P_1_ was less winter dormant than P_2_. P_1_ was evergreen, with expanded leaves and some shoot growth at the base of the plant in winter. P_2_ had rosette-like dormant shoots in winter. In spring 2016, 94 F_1_ plants were produced from a cross between P_1_ and P_2_. The morphology of F_1_ was similar to that of P_2_, but the leaf color, size, and shape, as well as plant height, varied.

P_1_ (4 plants), P_2_ (1 plant), and F_1_ (94 plants) were grown in a growth chamber and planted in pots (18 cm diameter, 14 cm height) around May 2017. The plants were placed in an experimental field of the Arid Land Research Center of Tottori University in Tottori, Japan (north latitude: 35.535, east longitude: 134.212) and grown throughout the year. Plants were irrigated immediately after planting and four times in June 2017 and once in July 2017. Plants were irrigated and fertilized twice in April 2018 and irrigated in August 2018. When multispectral images were collected to measure color changes (from 26 March 2019), only natural rainfall was allowed.

### 4.2. Multispectral Imaging and Analysis

The shooting dates (16 in total) were 26 March, 16 April, 2 May, 23 May, 13 June, 16 July, 26 August, 1 October, 31 October, 27 November, 16 December 2019, 11 January, 4 February, 6 March, 26 March, and 9 April 2020. No shooting took place in September 2019. Photographs were taken around noon, which is considered to be the least affected by sunlight. Tents were used to provide shade and to prevent direct sunlight from shining on the plants. A multispectral camera was placed directly above the plants, and each plant was photographed individually. Blue light (445 nm), green light (500, 532, 550, 568 nm), red light (676, 680, 700 nm), and near-infrared light (800 nm) were used. One image of each of the 99 individuals (4 P_1_, 1 P_2_, 94 F_1_) at each wavelength was manually selected from the shooting data. The plant part was extracted from each image, a binary image was produced, and the reflectance value at each wavelength was calculated. MATLAB (R2019b, MathWorks) was used for the data analysis up to this point. RStudio (version 3.6.2) was used for the subsequent data analysis [36]. The values were converted to ratios so that the sum of the nine values would be 1 and were used to calculate 15 vegetation indices related to biomass (5 indices), pigments (2), chlorophyll content (2), anthocyanins (3), carotenoids (2), and light use efficiency (1) (Table S2).

### 4.3. Genotyping and QTL Analysis

The P_1_, P_2_, and 94 F_1_ individuals were used to prepare a library for RAD-seq [20]. Stacks software was used to analyze the RAD-Seq reads as in [20,37]. QTL analysis was performed using the linkage map [20].

## 5. Conclusions

The quantitative measurements over time captured seasonal morphological changes in *P. takesimensis*. The vegetation indices and PCA analysis of reflectance at multiple wavelengths allowed us to capture seasonal changes. A particularly large number of QTLs was detected during dormancy breaking. Several chromosomal regions with high LOD values, although not exceeding the significance threshold, were found on several consecutive dates. Our approach could be used to capture seasonal changes in other plant species.

## Data Availability

The data are available from the corresponding author upon reasonable request.

## Supplementary Materials

The following supporting information can be downloaded from https://www.mdpi.com/article/10.3390/plants12203597/s1: Figure S1. Monthly changes in vegetation indices; Figure S2. QTL analysis on 13 June 2019; Figure S3. Seasonal changes in plant appearance; Table S1. Summary of the QTLs detected in this study; Table S2. Vegetation indexes and calculation formulas analyzed in this study.
