# Supplementary material for: Genetic Dissection of Seasonal Changes in a Greening Plant Based on Time-Series Multispectral Imaging
Source: Plants (Basel). 2023 Oct 17;12(20):3597. doi: 10.3390/plants12203597 (PMC10610531; doi:10.3390/plants12203597)
Supplement: Supplementary file 1 [file plants-12-03597-s001.zip › Figure_S1_20231017.pdf]

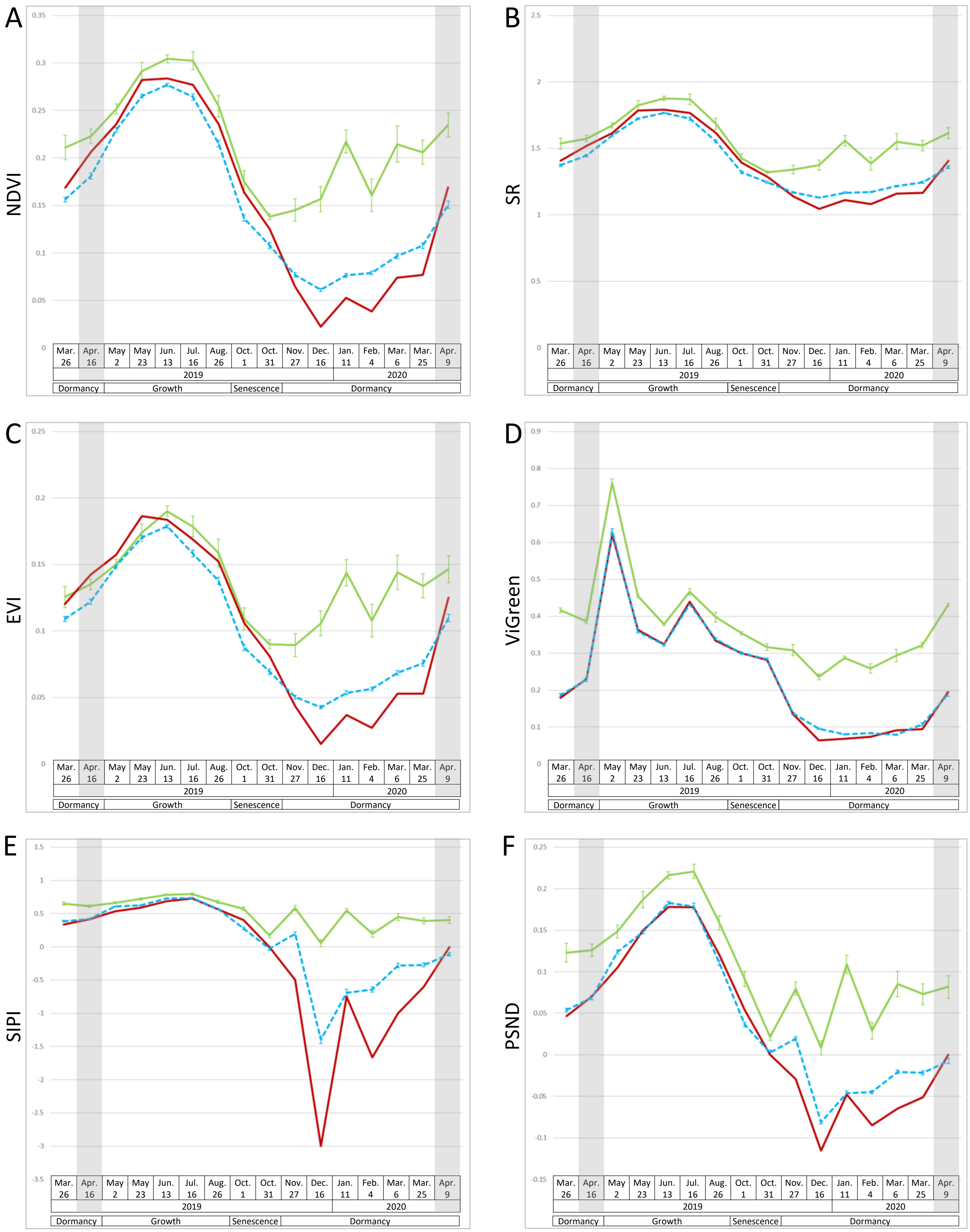
A
NDVI
0.35
0.3
0.25
0.2
0.15
0.1
0.05
0
B
SR
2.5
2
1.5
1
0.5
0
C
EVI
0.25
0.2
0.15
0.1
0.05
0
D
ViGreen
0.9
0.8
0.7
0.6
0.5
0.4
0.3
0.2
0.1
0
E
SIPI
1.5
1
0.5
0
-0.5
-1
-1.5
-2
-2.5
-3
-3.5
F
PSND
0.25
0.2
0.15
0.1
0.05
0
-0.05
-0.1
-0.15
Mar. 26
Apr. 16
May 2
May 23
Jun. 13
Jul. 16
Aug. 26
Oct. 1
Oct. 31
Nov. 27
Dec. 16
Jan. 11
Feb. 4
Mar. 6
Mar. 25
Apr. 9
2019
2020
Dormancy
Growth
Senescence
Dormancy

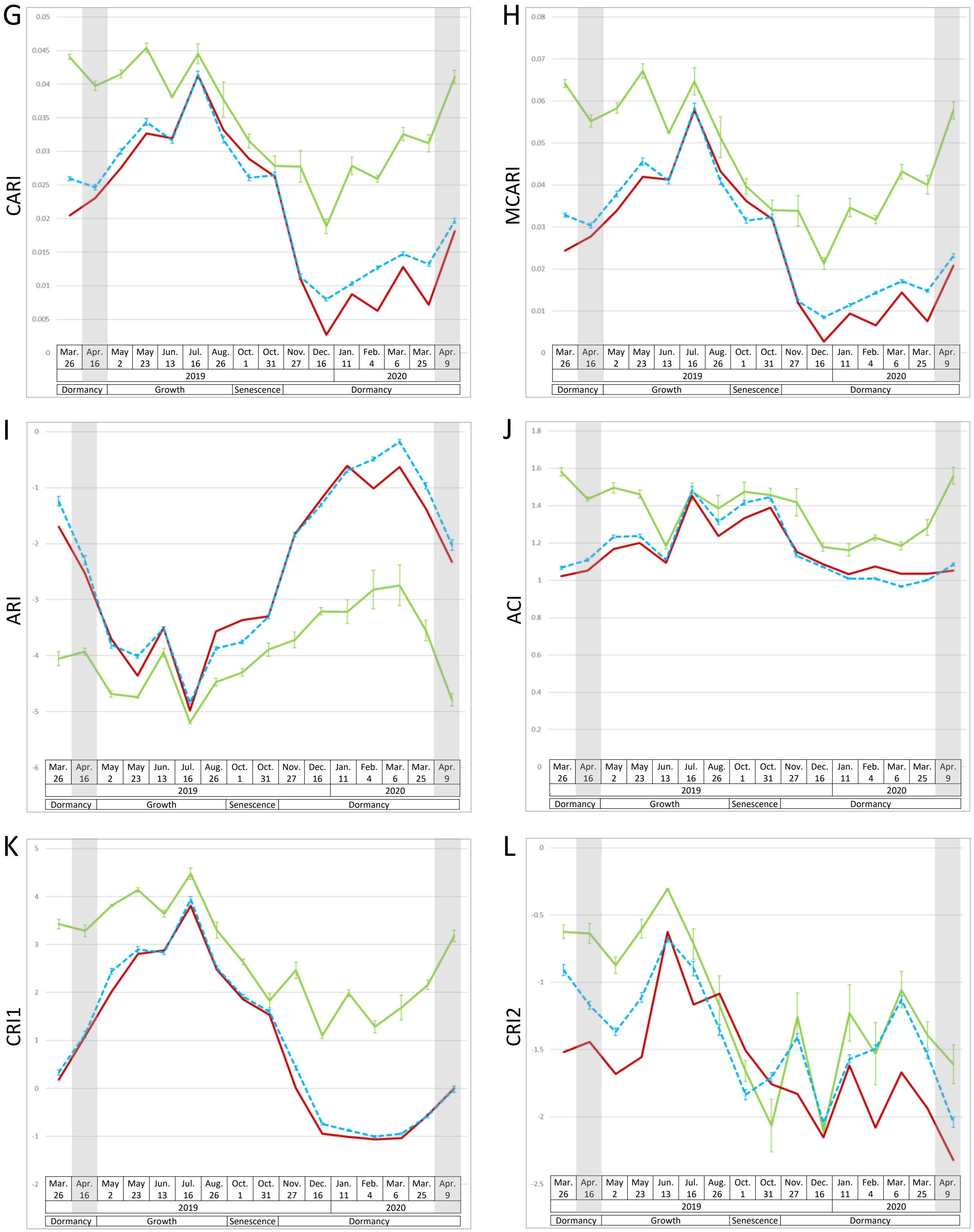

G
CARI
H
MCARI
I
ARI
J
ACI
K
CRI1
L
CRI2
Mar. 26
Apr. 16
May 2
May 23
Jun. 13
Jul. 16
Aug. 26
Oct. 1
Oct. 31
Nov. 27
Dec. 16
Jan. 11
Feb. 4
Mar. 6
Mar. 25
Apr. 9
2019
2020
Dormancy
Growth
Senescence
Dormancy

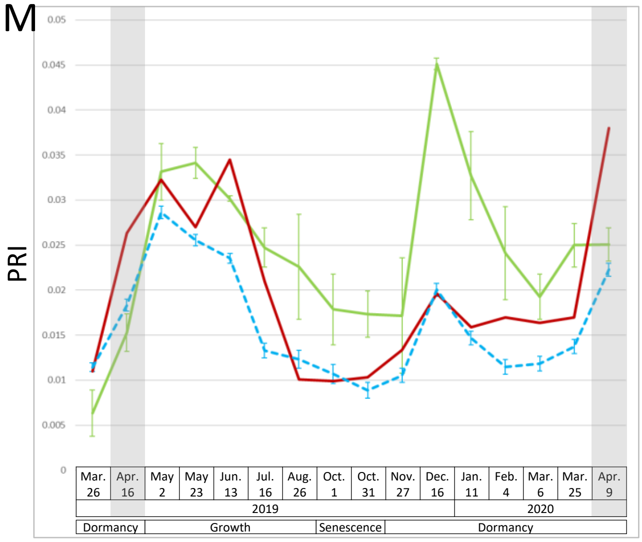

Figure S1.

Monthly changes in vegetation indices.

Indices are indicated in vertical axis labels. Green line, mean of P_1 (*n* = 4); red line, P_2 (*n* = 1); blue dashed line, mean of F_1 (*n* = 94). Error bars, standard errors. Gray shading, the dates used in [20].
