# Supplementary material for: Genetic Dissection of Seasonal Changes in a Greening Plant Based on Time-Series Multispectral Imaging
Source: Plants (Basel). 2023 Oct 17;12(20):3597. doi: 10.3390/plants12203597 (PMC10610531; doi:10.3390/plants12203597)
Supplement: Supplementary file 1 [file plants-12-03597-s001.zip › Figure_S2_20231016.pdf]

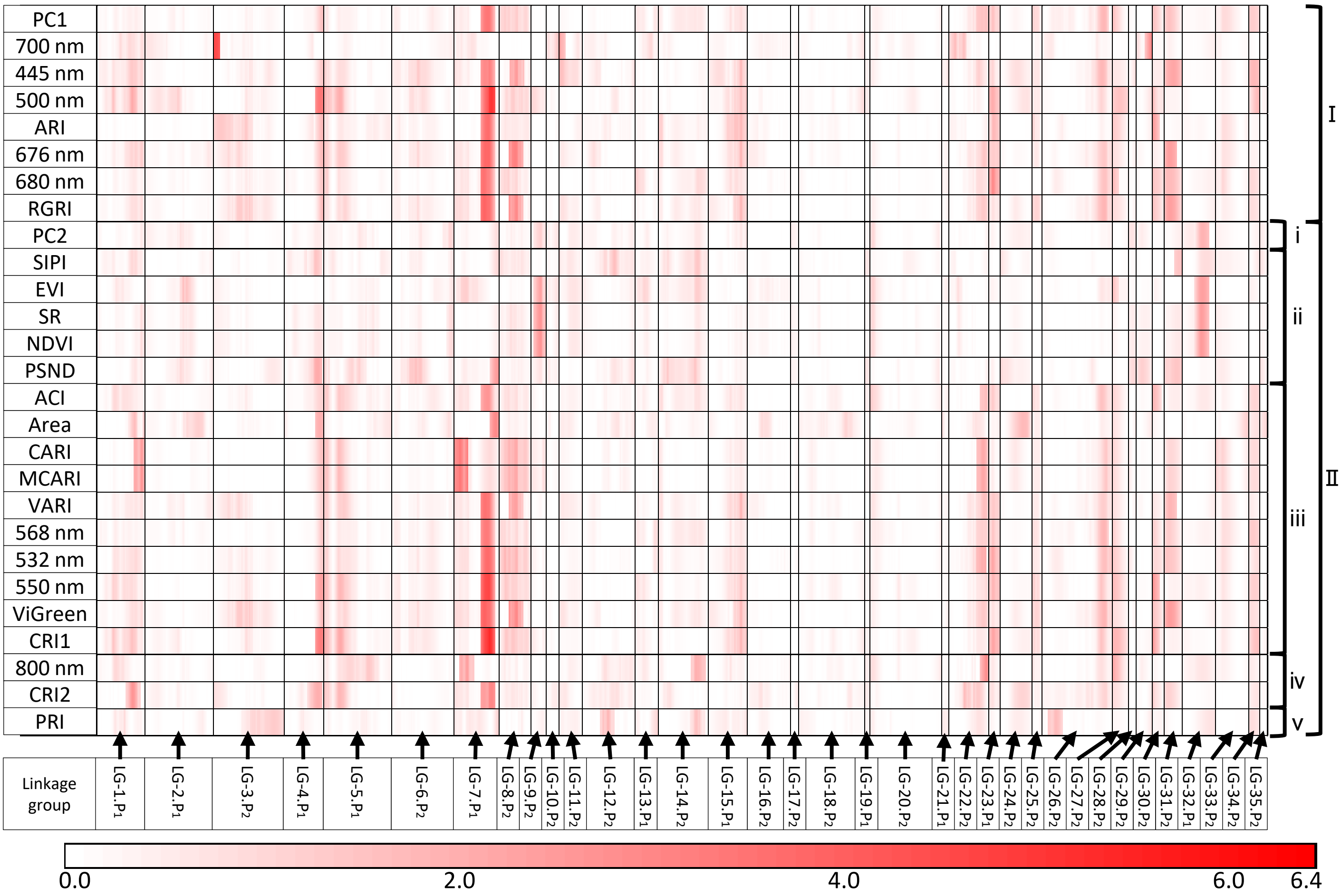

Figure S2.
QTL analysis on 13 June 2019.
Traits are listed along the vertical axis and the positions of chromosomal markers are indicated along the horizontal axis. Darker red indicates higher LOD values. Black arrows indicate each linkage group. The order of the traits and the Roman numerals on the right are as in Figure 3.
