# Supplementary material for: Genetic Dissection of Seasonal Changes in a Greening Plant Based on Time-Series Multispectral Imaging
Source: Plants (Basel). 2023 Oct 17;12(20):3597. doi: 10.3390/plants12203597 (PMC10610531; doi:10.3390/plants12203597)
Supplement: Supplementary file 1 [file plants-12-03597-s001.zip › Figure_S3_20231017.pdf]

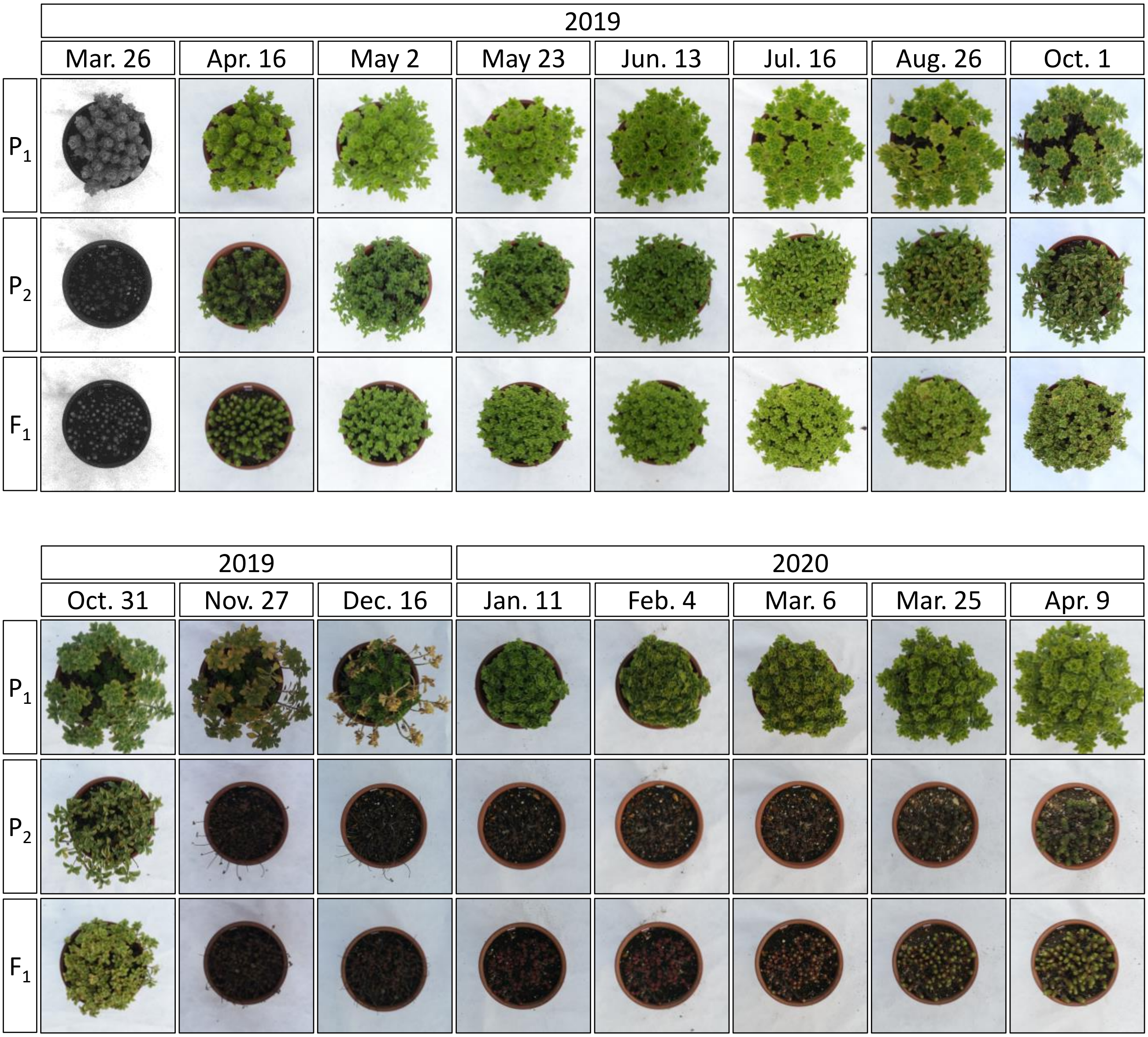

Figure S3.

Seasonal changes in plant appearance.

The photos (RGB images) show one of the four P_1 individuals, P_2, and one of the F_1 individuals. For March 26, 2019, a monochrome image with a reflection value of 550 nm was available.
